# Depressive symptoms, anxiety and cognitive impairment: emerging evidence in multiple sclerosis

**DOI:** 10.1038/s41398-023-02555-7

**Published:** 2023-07-19

**Authors:** Monica Margoni, Paolo Preziosa, Maria A. Rocca, Massimo Filippi

**Affiliations:** 1 Neuroimaging Research Unit, Division of Neuroscience, IRCCS San Raffaele Scientific Institute, Milan, Italy; 2 Neurorehabilitation Unit, IRCCS San Raffaele Scientific Institute, Milan, Italy; 3 Neurology Unit, IRCCS San Raffaele Scientific Institute, Milan, Italy; 4 Vita-Salute San Raffaele University, Milan, Italy; 5 Neurophysiology Service, IRCCS San Raffaele Scientific Institute, Milan, Italy

**Keywords:** Molecular neuroscience, Predictive markers

## Abstract

Neuropsychiatric abnormalities may be broadly divided in two categories: disorders of mood, affect, and behavior and abnormalities affecting cognition. Among these conditions, clinical depression, anxiety and neurocognitive disorders are the most common in multiple sclerosis (MS), with a substantial impact on patients’ quality of life and adherence to treatments. Such manifestations may occur from the earliest phases of the disease but become more frequent in MS patients with a progressive disease course and more severe clinical disability. Although the pathogenesis of these neuropsychiatric manifestations has not been fully defined yet, brain structural and functional abnormalities, consistently observed with magnetic resonance imaging (MRI), together with genetic and immunologic factors, have been suggested to be key players. Even though the detrimental clinical impact of such manifestations in MS patients is a matter of crucial importance, at present, they are often overlooked in the clinical setting. Moreover, the efficacy of pharmacologic and non-pharmacologic approaches for their amelioration has been poorly investigated, with the majority of studies showing marginal or no beneficial effect of different therapeutic approaches, possibly due to the presence of multiple and heterogeneous underlying pathological mechanisms and intrinsic methodological limitations. A better evaluation of these manifestations in the clinical setting and improvements in the understanding of their pathophysiology may offer the potential to develop tools for differentiating these mechanisms in individual patients and ultimately provide a principled basis for treatment selection. This review provides an updated overview regarding the pathophysiology of the most common neuropsychiatric symptoms in MS, the clinical and MRI characteristics that have been associated with mood disorders (i.e., depression and anxiety) and cognitive impairment, and the treatment approaches currently available or under investigation.

## Introduction

Multiple sclerosis (MS) is a chronic, inflammatory, demyelinating and neurodegenerative disease that affects the central nervous system (CNS), often leading to the accumulation of irreversible clinical disability [1].

During recent years, there has been an increasing understanding of MS pathophysiology which has influenced the development of novel treatment approaches. Beside locomotor disability, the growing evidence of neuropsychiatric symptoms occurrence in MS has provided new valuable insights in the pathophysiology of the disease, enabling novel therapeutic targets aimed to improve MS patients’ management [2, 3].

Among neuropsychiatric abnormalities, cognitive impairment, clinical depression, and anxiety are the most common with a higher frequency in MS patients compared to the general population [4].

It is now clear that the evaluation of physical disability should be combined with neuropsychological batteries and scales for a more detailed characterization of cognitive performance, depressive and anxiety symptoms, often neglected in the clinical practice, even though they have profound consequences on patients’ daily activities and quality of life (QoL).

In this scenario, the application of magnetic resonance imaging (MRI) measures, which are specific to different pathological substrates of MS, has consistently shown that, in addition to genetic, environmental and immunologic factors, structural and functional abnormalities in relevant brain regions and networks may contribute to identify the mechanisms of these heterogeneous manifestations of the disease [3]. Moreover, emerging findings suggest that specific pharmacologic and rehabilitative approaches can exert beneficial effects on cognitive functioning, mood disorders and fatigue, improving MS patient’s QoL.

In this review, we endeavor to provide a concise and updated overview regarding the most common neuropsychiatric symptoms of patients with MS, moving from pathophysiology to treatment approaches.

## Depressive and anxiety disorders

### Major depressive disorder

#### Epidemiological and clinical features

Major depressive disorder (MDD), also known as clinical depression, is a debilitating disease characterized by at least one major depressive episode lasting at least 2 weeks and involving clear-cut changes in mood, interests and pleasure, changes in cognition and vegetative symptoms [5]. Some symptoms are more specific to a depressive disorder, such as anhedonia (i.e., diminished ability to experience pleasure); diurnal variation (i.e., symptoms of depression are worse during certain periods of waking hours); and intensified guilt about being ill. Other symptoms, such as neurovegetative symptoms, including fatigue, loss of appetite or weight, and insomnia, are common also in other medical conditions [6].

If episodes of depression do not resolve and last for extended periods of time, this pattern is described as chronic depression. If depressive symptoms are present (on most days) for at least 2 years without any periods of remission exceeding 2 months, the condition is termed persistent depressive disorder or dysthymia [7].

The 12-month prevalence of MDD in general population varies considerably across countries but is approximately 6%, overall [8]. Interestingly, the 12-month prevalence of MDD is similar when comparing high-income countries (5.5%) with low- and middle-income countries (5.9%), suggesting that MDD is neither a simple consequence of modern day lifestyle in developed countries, nor poverty [9].

In MS, clinical depression is higher than in other neurologic disorders [10] and, depending on the reference point, is 3–10 times the rate in the general population [11].

According to a comprehensive systematic review [12], clinical depression represents the most prevalent comorbidity in MS (23.7%), followed by anxiety (21.9%), hypertension (18.6%), hypercholesterolemia (10.9%), and chronic lung disease (10%). A recent meta-analysis reported a higher prevalence of clinical depression in MS; the weighted prevalence of 58 studies on depression in MS was 30.5% (95% confidence intervals [CI] = 26.3–35.1%) [13].

Similarly to the general population, the prevalence peak is generally between 45 and 59 years in these patients [14]. Differently from other comorbidities, such as hypertension and hypercholesterolemia, the prevalence of clinical depression seems not increasing with age [15]. While some studies reported a higher prevalence of depressive symptoms in female MS patients with a history of depression [16–18], others did not [19].

Depressive symptoms typically associate with a progressive MS course, leading to severe consequences on cognitive performance and worsening physical disability [20–22].

Several scales have been proposed to score depressive symptoms in MS patients (Table 1). According to the evidence-based guidelines of the American Academy of Neurology (AAN) [23], the Beck Depression Inventory (BDI) scale is recommended for assessing depression in these patients [24]. It should be mentioned that this scale has to be adapted, since some questions overlap with disability. Other scales, including the BDI-II [25] and Montgomery-Asberg Depression Rating Scale (MADRS) [26], are commonly used in clinical practice (Table 1).

**Table 1:** Neuropsychological batteries and scales used to explore cognitive performance and to evaluate the presence of depressive symptoms and fatigue in patients with multiple sclerosis.

Cognition		
BRB-N [210]	Information processing speed	SDMT
	Working memory	PASAT 3s
	Verbal memory	SRT
	Visuospatial memory	SPART (10/36)
	Executive functions	WLG 90
	Verbal fluency	
BICAMS [211]	Information processing speed	SDMT
	Working memory	–
	Verbal memory	CVLT-II
	Visuospatial memory	BVMT-R
	Executive functions	–
	Verbal fluency	
MACFIMS [212]	Information processing speed	SDMT
	Working memory	PASAT 3s PASAT 2s
	Verbal memory	CVLT-II
	Visuospatial memory	BVMT-R
	Executive functions	COWAT
	Verbal fluency	D-KEFS sorting test
		JLO

Depressive symptoms	
Commonly used	Others
• BDI [24] • BDI-II [25] • MADRS [26] • HDRS [213]	• HADS [214] • CESD • PHQ-9 [215] • HSCL-25 [216] • IDS-SR [217] • DASS-21 [218]

Fatigue	
Commonly used	Others
• MFIS [219] • FSS [220]	• FAI [221] • FSMC [222] • RPE [223] • WEIMuS [224]

*BDI* Beck Depression Inventory, *BRB-N* Brief Repeatable Battery of Neuropsychological Tests in multiple sclerosis, *BVMT-R* Brief Visuospatial Memory Test, Revised, *CESD* Center for Epidemiologic Studies Depression Scale, *COWAT* Controlled Oral Word Association Test, *CVLT-II* California Verbal Learning Test, Second edition, *DASS-21* Depression Anxiety Stress Scale, *D-KEFS sorting test* Delis Kaplan Executive Function System sorting test, *FAI* Fatigue Assessment Instrument, *FSMC* Fatigue Scale for Motor and Cognitive Functions, *FSS* Fatigue Severity Scale, *HADS* Hospital Anxiety and Depression Scale, *HDRS* Hamilton Depression Rating Scale, *HSCL-25* Hopkins Symptom Checklist-25, *IDS-SR* Inventory of Depressive Symptomatology, *JLO* Judgment of Line Orientation test, *MACFIMS* Minimal Assessment of Cognitive Function in Multiple Sclerosis, *MADRS* Montgomery Asberg Depression Rating Scale, *MFIS* Modified Fatigue Impact Scale, *PASAT 2s* Paced Auditory Serial Addition Test 2.0s, *PASAT 3s* Paced Auditory Serial Addition Test 3.0s, *PHQ-9* Patient Health Questionnaire-9, *RPE* Borg Rating of Perceived Exertion, *SDMT* Symbol Digit Modalities Test, *SPART (10/36)* Spatial Recall Test, *SRT* Selective Reminding Test, *WEIMuS* Würzburg Fatigue Inventory for Multiple Sclerosis, *WLG 90* Word List Generation test.

Several studies showed that depressive symptoms severity was associated with fatigue, even after adjusting for disability status, and these conditions were significant and independent predictors of QoL in MS patients [20–22]. Depressive symptoms also related to cognitive functioning in MS since they negatively impact performance of attention, working memory, executive functions and information processing speed [27–30]. Indeed, MS patients with depressive symptoms have worse cognitive outcomes compared to HC but also to MS patients without depressive symptoms [22, 31, 32].

Suicide is a relevant consequence of depression with a prevalence reported up to 22.1% in MS [33], twice compared to the general population [34]. Risk factors for the development of suicidal ideation include current depressive symptoms, being female [35], young age at onset of MS, previous history of depression, social isolation, recent functional deterioration and abuse of illicit substance [36, 37].

#### Pathophysiology

The pathogenesis of MS-related depressive symptoms is multifactorial. Genetic, immunologic, structural and functional brain damage might contribute to the presence of such manifestations in MS patients [38].

Although the role of genetic factors in the pathogenesis of depression in MS has been poorly explored, a correlation between the presence of Apolipoproteinε2 allele and a decreased incidence of depression has been observed, suggesting a protective role of this allele [39].

Conversely, the harmful role of proinflammatory cytokines is consistent among different studies [40, 41]. Pro-inflammatory cytokines may affect serotonin synthesis and reuptake in the CNS, and, consequently, may lead to the malfunctioning of noradrenergic and serotoninergic circuits that represent the pathways targeted by several antidepressant drugs [42–44]. Specifically, interferon (IFN)-γ may induce tryptophan metabolism and higher blood cytokine levels, in particular interleukin (IL)-1, IL-6 and tumor necrosis factor-α, which might constitute the trigger for the increased secretion of adrenal corticosteroids through the activation of the hypothalamic–pituitary–adrenal (HPA) axis, linked to the onset of depressive symptoms [42–44]. In MS, immune system abnormalities are believed to occur before the onset of depression, but the suggestion that depression is always secondary to inflammation is controversial [40], because depression itself may compromise the immune system.

As discussed below, structural and functional brain abnormalities, especially involving fronto-temporal and limbic cortices, are also related to the presence and worsening of depression in MS patients. Notably, abnormalities in communication between key anatomical areas involved in modulation of mood (i.e., amygdala and ventrolateral prefrontal cortex) in relation to specific tasks were observed in MS patients, even in the absence of depression [45]. These findings might explain, at least partially, the high proportion of MS patients with depressive symptoms, as disconnection of a key mood-regulation pathway could compromise an individual’s emotional adaptability when confronted by the vicissitudes of life.

#### MRI findings

MS is classically characterized by the formation of macroscopic focal white matter (WM) lesions and diffuse damage to the so-called normal-appearing WM (NAWM) [1]. Besides, pathological and MRI studies have consistently shown the presence of abnormalities also affecting deep gray matter (GM) and cortex [1].

Compared to non-depressed, depressed MS patients showed higher brain T2-hyperintense lesion volume (LV) in the temporal lobe [46] and arcuate fasciculus [47, 48], and higher T1-hypointense WM LV in the superior frontal and parietal regions [49]. The described lesions were located at the projection areas of the basal limbic system [46], in line with neuropathological studies in depressed patients with Parkinson’s disease showing reduced neuronal cell count in nuclei associated with the limbic system, such as the raphe nuclei, the ventral segmental nuclei or the locus coeruleus [50, 51]. Notably, a similar localization of WM lesions was also observed in depressed patients with vascular pathology, suggesting a common pathogenesis of secondary depression [52].

Recently, advanced MRI techniques have shed light on brain microstructural abnormalities associated with depression in MS patients. A lower NAWM fractional anisotropy and a higher mean diffusivity in the NAGM in the temporal lobe and in inferior frontal regions, and atrophy of cortical regions located in the bilateral frontal lobes were found in MS patients with depression compared to those without depression [53]. Atrophy of cortical regions located in the bilateral frontal lobes and entorhinal cortex and cerebellum were also significant predictors of depression [54–56]. An involvement of hippocampus has also been described in depressed-MS patients, as demonstrated in a study where hippocampal atrophy correlated with the severity of depression [57].

Moreover, structural connectivity abnormalities between the right hippocampus, right amygdala and frontal regions were associated with the presence of depression, suggesting that connectivity alterations at the limbic-motor interface may explain the occurrence of depression in MS patients [58].

Functional MRI (fMRI) studies showed an increased activity of the ventrolateral prefrontal cortex, with, however, a trend to hypoconnectivity with the amygdala and the medial prefrontal cortex in MS patients with depression. This is possibly related to a maladaptive emotional coping that might cause a higher vulnerability to depression of MS patients [45].

When evaluating resting state (RS) fMRI, a functional disconnection of the hippocampus with regions of the default mode network due to the accumulation of focal WM lesions has been found to be associated with depression [59], as well as an imbalance in the RS FC of the salience network, executive control network in cognitively preserved MS patients with depression [60]. Of note, these abnormalities were also observed in MS patients with cognitive impairment, possibly reflecting a common pathophysiology leading to an overlapping symptomatology (e.g., concentration and memory difficulties).

Recently, the potential contribution of HPA axis on depression in MS patients has been explored with controversial results [61, 62]. Proton magnetic resonance spectroscopy revealed increased glutamate levels in the hypothalamus of MS patients with depression, possibly reflecting a metabolic involvement of this structure in depression-related processes [61]. Another study showed that MS-related depression was associated with more distributed abnormalities involving the three explored monoaminergic networks (i.e., dopamine, norepinephrine and serotonin transporters), resulting in overall reduced RS FC in the frontal lobe, limbic areas and the precuneus [62]. Conversely, no significant associations between GM atrophy and atlas-based distribution of the main neurotransmitters (i.e., serotonergic, dopaminergic, noradrenergic, cholinergic and glutamatergic maps) were found for depression in a recent study [63]. These discrepancies may be related, at least partially, to the different methodologies applied (structural vs fMRI), as well as different MS populations investigated.

#### Therapeutic approaches

Diagnosis and treatment of mood disorders are fundamental to improve MS patient’s daily-life activities, QoL, as well as therapeutic compliance and adherence. Treatment of depression should be individualized and involve an association between pharmacological and non-pharmacological treatments.

A Cochrane review, published in 2011 [64], selected only two controlled double-blind randomized trials (RCTs). Desipramine (a tricyclic antidepressant) at the highest dosage of 200 mg/day [65] and paroxetine (a selective serotonin reuptake inhibitor) at a dosage of up to 40 mg/day [66] were compared with placebo in 32 and 42 depressed-MS patients, respectively on a 5- and 12-week period. In both trials, a trend towards efficacy was observed, although not reaching statistical significance.

In the absence of newer RCTs or observational studies in MS, treatment should follow the same guidelines as for the general population. First-line treatments of depression comprise serotonin reuptake inhibitors, followed by serotonin-norepinephrine reuptake inhibitors, such as venlafaxine and duloxetine, tricyclic antidepressants and mirtazapine [67].

Psychotherapy has long been considered an important treatment option for the management of depression in MS patients, with approaches focusing on coping skills showing superiority over insight-oriented therapies [68]. In this view, cognitive behavior therapy can help maximize the development of the patients’ coping skills [68]. The effectiveness of mindful-based intervention which is based on the nonjudgmental awareness of everyday moments has also been recently demonstrated [69]. However, these approaches cannot be used in cognitive impaired MS patients [69].

Regarding non-pharmacological treatments, transcranial magnetic stimulation is a recognized technique for the treatment of cognitive and mood symptoms in depression [7].

Although no data are available for the treatment of MS depressive symptoms, by applying lesion network mapping, a recent study found that MS lesions associated with depression are preferentially connected to the same circuit as stroke lesions, transcranial magnetic stimulation and deep brain stimulation sites that modify depression severity [70]. This supports not only that MS depression is associated with lesion location, but also that MS depression may share some neuroanatomical features with other depression etiologies and therapeutic neuromodulation sites.

The association between disease modifying treatments (DMTs) and mood disorders has been partially investigated. Early reports suggested an association between IFN-α, IFN-β and depression [71]. While a randomized controlled trial comparing IFN-β and glatiramer acetate showed no statistical differences between the two treatment groups in terms of BDI scores [72], the EPOC (Evaluate Patient Outcome) study showed that after switching from injectable medications (i.e., IFN-β and glatiramer acetate) to fingolimod BDI-II scores significantly reduced over a 6-month period [73].

Some observational studies suggested a positive effect of natalizumab and fingolimod on depression [74, 75], whereas the possible interplay between depression and other DMTs such as dimethyl fumarate, teriflunomide, alemtuzumab and ocrelizumab has not been studied yet.

### Anxiety disorders

#### Epidemiological and clinical features

Anxiety disorders comprise separation anxiety and selective mutism, which occur primarily in childhood, specific phobias, social anxiety disorder, generalized anxiety disorder, as well as panic disorder and agoraphobia, occurring primarily in adulthood [76].

Individuals with anxiety disorders are excessively fearful, anxious, or avoidant of perceived threats in the environment or internal to oneself [77]. The response is out of proportion to the actual risk or danger posed. Fear occurs because of perceived imminent threat whereas anxiety is a state of anticipation about perceived future threats. Panic attacks feature prominently as a particular type of fear response. Avoidance behaviors range from refusal to enter situations to subtle reliance on objects or people to cope [77].

Anxiety disorders are common symptoms in MS, with an age-standardized prevalence reported up to 35.6% (95% CI = 33.7–37.7%) of patients compared to 29.6% (95% CI = 28.8–30.5%) in the general population [14]. MS patients experience anxiety disorders at some point during their lives, however the prevalence peak is 45–59 years both in MS and in the general population [13, 14]. Anxiety disorders are often related to female sex, a younger age [78], and a closer MS onset and diagnosis [79]. Indeed, MS diagnosis could be a risk factor to develop anxiety disorders as the prevalence of self-reported anxiety symptoms at time of MS onset is 2.7%, whereas it becomes 6.2% by the time of diagnosis [80].

Several studies investigated the association between anxiety and cognitive functioning in MS [80–82], showing an association between a worse performance on executive functioning, visual memory, and information processing speed and a higher level of anxiety [81–83]. Moreover, MS patients with anxiety disorders experience significantly more fatigue, pain and sleep problems, which worsen with the co-occurrence of depression [84].

#### Pathophysiology

The pathophysiology of anxiety disorders is poorly understood in the general population, even less is known in MS. In the general population, genetic-epidemiological studies showed a moderate familial aggregation for anxiety disorders, with heritability estimates in the range of 30–50% [85].

As discussed below, several brain regions have been implicated in the modulation of anxiety disorders both in general population and MS, including amygdala, hippocampus and medial prefrontal cortex (the ventromedial prefrontal and anterior cingulate cortices). The involvement of hypothalamus, midbrain and brainstem has also been described.

Anxiety symptoms and the resulting disorders are thought to be due to disrupted modulation within the CNS. Several neurotransmitter systems have been implicated to have a role in one or several of the modulatory steps involved. A reduced activation of the serotonergic system and an over activation of the noradrenergic system have been described [86], resulting in dysregulation of physiological arousal and the emotional experience of this arousal [86].

The HPA axis has also been implicated in the pathogenesis of anxiety disorders. Reduced circulating cortisol levels and glucocorticoid hypersensitivity have been described in post-traumatic syndrome disorder [87]. Experimental studies have also showed that the HPA axis is hyperactivated in a wide range of models of stress and anxiety [88]. In turn, these findings support the role of glucocorticoids as crucial mediators of functional and anatomical abnormalities observed in cortical and limbic regions (acting through glucocorticoid and mineralocorticoid receptors) [89].

#### MRI findings

A few studies have investigated the association between anxiety symptoms and measures of brain structural and functional damage in MS patients with inconclusive results, reflecting the complexity of the disease.

Early studies showed no correlation between anxiety severity score and brain T2-hyperintense, T1-hypointense and gadolinium-enhancing WM lesions [90–92]. Conversely, more recent evidence revealed that MS patients with fatigue and anxiety symptoms had larger caudate volumes and a thinner left parietal cortex compared to those without fatigue; another study showed that MS-related anxiety may have its neuropathological substrate in the septo-fornical area [93]. The lack of definite pathological substrates leads to consider anxiety as a reactive response following disease progression [91]. However, a recent study showed that MS patients with higher anxiety severity score had increased atrophy in the ventrolateral prefrontal cortex, a crucial area for top-down control for threat and emotional processing [94], supporting a direct link between anxiety symptoms and structural damage [95].

Although anxiety has not been investigated in depth as depression in MS, several studies performed in people with generalized anxiety disorder showed an involvement of specific brain regions. A large meta-analysis [96] revealed that only atrophy of the anterior cingulate and inferior frontal cortex was associated with anxiety symptoms in patients with anxiety disorders compared to healthy controls. Atrophy of the ventromedial prefrontal cortex, a region associated with emotion and reward in decision-making, has been also detected in patients with generalized anxiety disorders [97].

Diffusion tensor MRI studies also revealed widespread abnormalities in regions involved in the generation and regulation of emotion, such as amygdala [98], uncinate fasciculus and cingulum in people with generalized anxiety disorder compared to healthy controls [99, 100]. Notably, reduced fractional anisotropy values in right uncinate fasciculus and left cingulum bundle showed significantly negative correlations with anxiety severity score, supporting the involvement of these structures in anxiety disorders [100].

A recent RS FC study performed in MS patients identified an anxiety-related network, comprising bilateral prefrontal cortex, amygdala and hippocampus, which correlated to atrophy of the dorsal pre-frontal cortex [95]. Interestingly, this network resembles previously observed patterns of network-level dysfunction described for generalized anxiety disorders [101]. It is tempting to speculate that the atrophy of the pre-frontal cortex alters the functional connectivity to specific brain areas (i.e., amygdala and hippocampus) distal from the primary spot of atrophy leading to the loss of information input from a damaged part of the brain [102].

Notably, in a recent meta-analysis including structural and functional MRI studies in generalized anxiety disorders, a reduced functional connectivity between pre-frontal cortex and amygdala was found resulting from tasks investigating emotion dysregulation [103].

#### Therapeutic approaches

Although a few clinical trials have been performed in MS patients with anxiety, pharmacological and non-pharmacological treatments are similar to those administered in general population [104]. Indeed, so far, no controlled studies on the effectiveness of psychological or pharmacological treatments of anxiety disorders were performed in MS patients, suggesting a need for research in this area. Only three clinical trials for depression evaluated the beneficial effect of psychological treatment on MS-related anxiety, without showing any statistical improvement, probably due to the co-occurrence of depressive symptoms [104].

A few RCTs and observational studies evaluated the effect of DMTs on anxiety symptoms. An improvement of anxiety symptoms was observed following both natalizumab and fingolimod treatment, which was not statistically significant [105–107].

## Cognitive impairment

### Epidemiological and clinical features

Cognitive impairment is a major cause of disability in MS with a prevalence ranging between 34 and 91% according to the cohorts of patients investigated, the neuropsychological tests used, and the criteria applied to define cognitive impairment, as explained further below [108–111].

Although the pattern of cognitive deficits is highly variable among MS patients, information processing speed, attention, learning, and memory are the most frequently involved domains, whereas deficits in executive functions and visuospatial processing are also reported, but less frequently [2, 3, 32].

Sex may influence cognitive functions in MS [112–114]. Compared to females, male MS patients seem to be more impaired on several cognitive domains, including verbal memory, executive functions, attention, memory, visuospatial processing, and information processing speed [112–114].

Cognitive impairment has been described in 20–25% of patients with clinically isolated syndrome (CIS), 30–45% of patients with relapsing-­remitting (RR) MS, and 50–75% of patients with secondary progressive (SP) MS [115]. Although the prevalence of cognitive impairment in primary progressive (PP) disease varies greatly, depending on the population considered, it occurs in up to 91% of patients [108]. Cognitive dysfunction has been also described in subjects with a radiologically isolated syndrome [RIS] [116], where MRI findings suggestive of MS are incidentally found in an asymptomatic subject. In particular, cognitive deficits can precede the appearance of other neurological symptoms and signs and are associated inflammatory-demyelinating lesions of the CNS seen on MRI [109, 116]. Cognitive deficits have been also found in more than 50% of patients with pediatric-onset MS, i.e., in those patients where the clinical onset of the disease occurs before the age of 18 years. These patients are characterized by worse performance in information processing speed and memory as well as verbal intelligence compared to age-matched healthy controls [117]. Decreased intelligence quotient and academic skills have also been described [118]. It is likely that inflammation of the brain during critical developmental periods [119], including myelinogenesis in adolescence, may irreparably damage neural networks involved in such domains [120].

Several test batteries have been recommended and validated to explore cognitive performance in MS patients [121]. While the more lengthy and complex Brief Repeatable Battery-Neuropsychology (BRB-N, 45 min) and MACFIMS (Minimal Assessment of Cognitive Function in Multiple Sclerosis, 90 min) are usually applied in research settings, a shorter assessment, such as the Brief International Cognitive Assessment for multiple sclerosis (BICAMS) [122], or the assessment of only information processing speed using the Symbol Digit Modalities Test (SDMT) [123], may be more appropriate for clinical use (Table 1). While MACFIMS has a stronger psychometric foundation compared to BRB-N and includes assessment of spatial processing and higher executive function abilities, both tests provide information regarding working memory, executive functions and verbal fluency compared to BICAMS (Table 1).

Interestingly, some longitudinal studies with a long follow-up provided important insights into the pattern of cognitive evolution in MS. Two of these studies observed deterioration in simple and complex auditory attention span and episodic verbal learning and memory, with one showing additional worsening in visuospatial memory, whereas the other found additional deterioration in information processing speed and visual construction [124, 125]. The third study showed significant deterioration only in information processing speed and complex attention [126].

Recently, the traditional dichotomous classification of cognitive functioning, namely, preserved *vs* impaired cognition, has been challenged. Indeed, by applying different methodologies, such as machine learning or more classification-style approaches (e.g., International Classification of Cognitive Disorders in Epilepsy; IC-CoDE), recent studies have examined cognitive impairment in MS by identifying distinctive cognitive phenotypes [127–130]. These studies identified from three to five phenotypes, highlighting a spectrum of cognitive function ranging from intact to a multi-domain impairment [127–130]. Compared to the dichotomous classification of cognitive impairment, the definition of specific cognitive phenotypes may represent a step toward tailored treatment approaches and toward improving understanding of the different pathophysiological mechanisms related to cognitive changes in MS.

Notably, the revised fifth edition of Diagnostic and Statistical Manual of Mental Disorders (DSM-5) proposed a common framework for the diagnosis of neurocognitive disorders, including mild and major neurocognitive disorders [131]. Briefly, the DSM-5 diagnosis of major neurocognitive disorder requires substantial impairment to be present in one or (usually) more cognitive domains, and this must be sufficient to interfere with independence in everyday activities. The diagnosis of mild neurocognitive disorder is made when there is modest impairment in one or more cognitive domains and the individual is still independent in everyday activities, albeit with greater effort. The impairment must represent a decline from a previously higher level and should be documented both by history and by objective assessment. Further, the cognitive deficits must not occur exclusively in the context of a delirium or be better explained by another mental disorder.

The DSM‐5 classification was designed to complement the clinical process in which a diagnosis is made in two steps: a syndromal diagnosis is made first, and then potential causative factors are examined to attribute etiology [131]. Although mild and major neurocognitive disorders are subtyped according to their etiology, MS is not included in these categories yet.

Even if effort is ongoing to derive consensus-based assessment approaches to define cognitive impairment in MS, there is a disconnection between research and clinic in MS that has yet to be solved.

### Pathophysiology

**Fig. 1:**
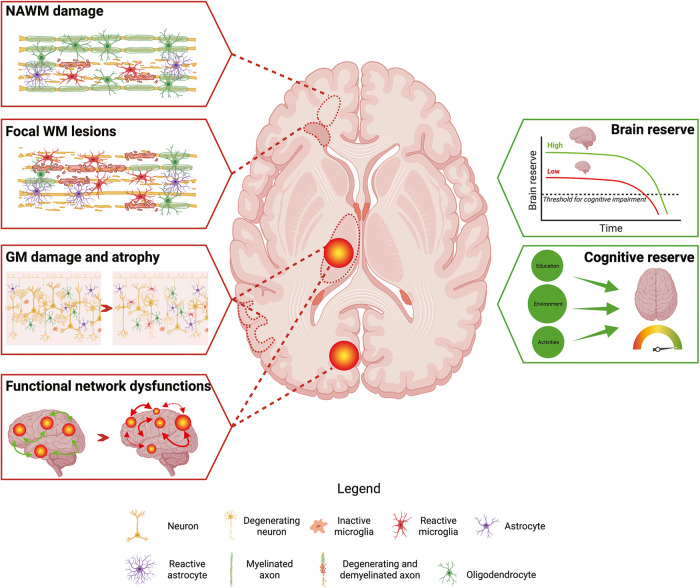
Schematic representation of the main substrates underlying cognitive impairment in multiple sclerosis. WM and GM damage (i.e., focal lesions and subtle abnormalities in normal-appearing brain tissues), and functional abnormalities, especially in strategic regions, can explain the presence and severity of cognitive impairment. Brain and cognitive reserve may counterbalance these detrimental processes. See text for further details. Created with biorender.com. GM gray matter, NA normal appearing, WM white matter.

Owing to its sensitivity and specificity toward MS-related abnormalities, MRI has been widely applied to improve the understanding of the mechanisms related to the occurrence and accumulation of cognitive deficits in MS patients. Structural abnormalities of brain WM and GM and functional alterations of brain networks, especially in strategic regions, may contribute to the presence and severity of cognitive impairment through a primary GM damage or through a disconnections of cognitively-relevant brain regions (Fig. 1) [3].

Although the MRI evaluation of WM and GM damage has improved the ability to predict cognitive outcomes in MS, a discrepancy between the disease burden (e.g., brain WM lesions) and cognitive performance still exists, whereby some patients have better preserved cognition that others despite similar WM lesion volume. This emphasizes the role of additional factors, including brain reserve and cognitive reserve [3].

The brain reserve hypothesis states that people with higher brain reserve withstand more severe disease burden before experiencing cognitive decline [132]. Brain reserve is attained during the critical period of the human development and refers to structural characteristics (i.e., maximal lifetime brain growth) [132]. Intracranial volume (ICV) is an estimate of maximal lifetime brain growth, as brain growth corresponds to increased ICV during development [133]. MS patients with higher ICV showed better cognitive scores in the SDMT and Paced Auditory Serial Addition Task-3 (PASAT-3) measures compared to those with lower ICV [134]. Of note, this protective effect was specific for these cognitive domains and was not related to memory functions [134].

The cognitive reserve hypothesis [132, 134, 135] relies on the evidence that daily-life activities and increased intellectual enrichment, such as vocabulary, literacy, intelligence, education, work and engagement in cognitive enriching leisure activities, may mitigate the negative effect of disease-related structural damage on cognitive status, particularly memory abilities [134, 136–139]. A protective effect was observed for life experience (i.e., early life cognitive leisure) and education, independently from ICV [134]. Different studies showed that both RRMS [140, 141] and SPMS patients [142] with higher intellectual enrichment were less likely to suffer disease-related cognitive impairment. Notably, the potential protective role of cognitive reserve against cognitive dysfunction was also evident in pediatric MS [143]. In these patients, higher intelligence quotient scores were associated with stable or improved cognitive performance at subsequent evaluations, particularly in cognitively-preserved subjects at the first assessment [143]. These positive effects appeared to be maintained during adulthood [144, 145]. Therefore, cognitive reserve could be particularly efficient in children, who may have greater capacity to compensate from brain damage through neural plasticity.

In this view, cognitive impairment is more prevalent in older MS patients compared to younger patients [146]. Some evidence also suggested that male patients had more vulnerability to cognitive deficits compared to female patients in MS [147].

Since cognitive reserve is potentially a modifiable factor, its enhancement through physical exercise, mentally active lifestyles, management of cardiovascular risk factors and other comorbidities, might be a therapeutic target to prevent or slow cognitive deterioration in MS patients [148].

### MRI findings

#### Structural brain MRI

Disconnection mechanisms have been suggested to contribute to cognitive impairment in MS patients, through the disruption of integration between different cognitively relevant brain regions [3].

Consistently with this hypothesis, correlative clinical–lesional studies have demonstrated that brain T2-hyperintense WM LV and the location of lesions in cognitively-relevant WM tracts or regions help to explain global cognitive dysfunction as well as deficits in specific cognitive domains in MS patients [3, 149, 150]. A structural connectivity disruption was observed in RRMS patients within visual network, between visual and deep GM networks, and between default mode and frontoparietal networks, and correlated with worse working memory [151]. In another study, a close correlation between lower information processing speed and higher structural disconnection in the default mode network was found in RRMS patients with and without cognitive impairment, being more pronounced in the former group [152]. Worse executive control in RRMS patients correlated with higher structural disconnection in the frontoparietal networks, deep GM structures and insula [153], and within sensorimotor, dorsal attention, left frontoparietal, and default mode networks [154].

Moreover, the severity of lesional microstructural abnormalities in WM tracts that are critical for cognitive functions, such as the cingulum, were also found to be relevant predictors of global cognitive impairment and of deficits in single cognitive domains [155].

In addition to focal WM lesions, diffuse NAWM damage may also undermine the physiological connections among cognitively-relevant GM regions, contributing to a disconnection syndrome. Using diffusion tensor MRI, performance at global cognitive functions and at specific cognitive domains, including information processing speed, attention, memory, verbal fluency and executive functions, have been consistently found to be associated with microstructural abnormalities in relevant WM tracts, such as the corpus callosum, cingulum, fornix and thalamic connections [155–166]. Interestingly, these abnormalities partially overlapped with brain T2-hyperintense WM lesions, supporting an independent role of NAWM damage in determining cognitive impairment [162, 163, 165].

Focal and diffuse GM damage has been consistently identified among the best predictors of cognitive deficits. Using double inversion recovery (DIR) sequence, higher number and volume of cortical lesions, especially in the hippocampus, were found to be significantly associated with the severity of cognitive dysfunction [167–169] and to predict worsening of cognitive performance over 5 years [170]. Moreover, more severe atrophy and microstructural abnormalities of cognitively-relevant GM regions such as the thalamus, hippocampus, cortex and cerebellum have been consistently identified among the best contributors of worse cognitive performance in MS patients with the main disease clinical phenotypes [171–176], and to predict cognitive deterioration over up to 13 years [177, 178].

A few studies have analyzed the relationship between distinct cognitive deficits (e.g., memory or visuospatial impairment) and regional cortical atrophy in MS. These studies usually included relatively small samples or focused on specific tests or brain regions, with conflicting results. While a study showed no significant correlation between SDMT and regional brain volumes [179], more recent studies revealed an association with prefrontal cortex, precentral and postcentral gyri, and right temporal cortex [180] as well as thalamus, cerebellum, putamen, and occipital cortex [181].

So far, a few studies have investigated the differences of structural brain damage between males and female MS patients in cognitive impairment. Some studies suggested that more severe NAWM microstructural abnormalities [112] and subcortical GM atrophy [113] in male compared to female MS patients are two relevant pathological substrates contributing to sex-related differences in cognitive impairment in MS. In a recent study [182], worse cognitive performance seemed to be associated mainly with deep GM volume loss in female MS patients, and with cortical GM volume loss in male MS patients.

#### Functional brain MRI

Beside structural damage, fMRI studies may provide relevant pieces of information about the substrates underpinning cognitive impairment. MS patients without cognitive impairment have consistently shown increased and more widely distributed cortical recruitment than healthy controls during the performance of cognitive tasks [158, 183, 184]. These functional abnormalities are associated with measures of brain structural damage (i.e., brain T2-hyperintense WM LV, NAWM and GM damage) [158] and suggest that the increased activation during a task of crucial cortical regions/networks might represent adaptive processes able to attenuate the negative effect of MS-related tissue damage on cognitive function. However, such increased cortical recruitment cannot persist indefinitely, and the loss or exhaustion of adaptive mechanisms might contribute to cognitive decline [185–187].

Another mechanism that has been disclosed in MS patients when applying active fMRI tasks is the inability to optimize cognitive network recruitment with increasing task difficulty, which results from an impaired functional reserve (the ability to match brain activity to increasing cognitive demand) [188, 189]. This maladaptive mechanism contributes to the clinical manifestations of the disease, is more pronounced in patients with SPMS [189] and in those with cognitive impairment [190].

The network compensation-collapse hypothesis has been proven also using an analysis of RS FC. In the earliest phases of the disease and in MS patients without cognitive impairment, better cognitive performance was associated with increased RS FC among several regions of several brain networks, such as the attention network [191, 192]. On the other hand, in MS patients with cognitive impairment, more heterogeneous and inefficient patterns of RS FC abnormalities have been found. Indeed, reduced RS FC of anterior regions of the brain, mostly located in the frontal lobes [193, 194], associated with more severe cognitive impairment and with structural disruption of the connecting WM tracts [193], but also increased RS FC associated with worse cognitive performance [195, 196].

Cognitive functions are complex brain processes based on local processing and effective integration among different regions. Accordingly, pathological processes can determine cognitive dysfunction through a direct involvement of GM regions relevant for cognitive functions and the disruption of their connections. Consistently with this hypothesis, multiparametric MRI studies have further supported a complimentary and independent contribution of focal WM lesions, NAWM microstructural damage and GM atrophy, combined with functional brain network abnormalities, to cognitive impairment in MS patients [164, 165, 197–200]. At present, only two longitudinal studies [187, 201] have employed both structural and functional MRI techniques, finding an association between cortical [201] and deep GM atrophy, maladaptive excessive and inefficient recruitment of brain networks and worse cognitive performances over time [187, 201].

Recent work also pointed out an involvement of specific neurotransmitter systems to explain cognitive impairment in MS patients. In line with this, compared to cognitively preserved MS patients, those with cognitive impairment showed significant GM atrophy that was spatially correlated with a higher atlas-based distribution of specific receptor or transporter of dopamine, noradrenaline, acetylcholine and glutamate. Although these studies did not directly explore the impairment of specific neurotransmitters networks, these results suggested that cognitive impairment may be associated with a pattern of GM atrophy that is not random and involves regions with a high and specific distribution of neurotransmitters that are well-known to be involved in cognitive functions [63].

### Therapeutic approaches

By limiting disconnection mechanisms and atrophy, evidence from RCTs and observational studies supports the beneficial effects of DMTs not only on locomotor functions, but also on cognition in MS patients [202]. Notably, such beneficial effects may occur also in MS patients with a progressive disease course and more severe cognitive impairment, suggesting that, beyond their effect in reducing disease activity, DMTs can improve or, at least stabilize, cognitive functions [202].

Cognitive rehabilitation [203–205] and symptomatic treatments [206] may also contribute to preserve and improve cognitive functions in MS patients.

Cognitive rehabilitation typically refers to training targeting improvement of skill by regaining (re-establishing or strengthening) abilities that were intact prior to the loss. The other focus of cognitive rehabilitation is developing compensatory strategies for lost abilities when they cannot be regained. In contrast, the term cognitive intervention refers to targeted training of a particular cognitive skill or domain for the purpose of enhancement regardless of the baseline state of cognitive abilities [203]. Historically, most of the measures implemented for use involved learning and memory-based interventions, but recently the focus has moved to executive function and attention, since these are the cognitive domains most affected in MS [203].

Conflicting findings about the effectiveness of the various cognitive rehabilitation techniques exist and therefore no definite conclusions can be drawn about their effect on cognition. Two Cochrane reviews assessing 20 randomized controlled trials of behavioral interventions [205] and 15 interventional trials pointed out the low beneficial effects of rehabilitation on cognitive functioning. A separate systematic review evaluating 33 original intervention studies supported similar conclusions [203]. However, these findings may be limited by the evaluation of small samples of subjects or methodological biases; thus, future research should be devoted to better understating the potential benefits of such therapies. It is worth mentioning that a randomized clinical trial with a combined approach of cognitive rehabilitation and aerobic exercise is ongoing [207].

Similarly, at present there is insufficient evidence to support the use of symptomatic pharmacologic treatments (i.e., donepezil, rivastigmine, memantine) to improve cognitive function in people with MS [206, 208].

However, a multiparametric combined approach including DMTs, symptomatic therapies, rehabilitation together with the adoption of a healthy lifestyle (i.e., physical exercise, mental activity, prevention of cardiovascular disease and other comorbidities, smoking cessation, etc.) may be the most rewarding strategy to preserve cognitive integrity and to recover cognitive functions in MS patients showing cognitive deficits [148].

## Conclusions and future directions

Neuropsychiatric symptoms are frequently reported in MS patients. Among them, cognitive impairment, clinical depression and anxiety are the most common with a higher frequency in MS patients compared to the general population [4]. These symptoms may develop even before the diagnosis of MS [209], and, hence, early diagnosis is crucial to prevent complications. Although an improved understanding of the pathophysiological mechanisms underlying these conditions has led to the development of several neuropsychological batteries and scales to better identify these symptoms, definite treatment guidelines still not exist in MS.

Further studies are needed to clarify the complex interplay between MS and neuropsychiatric disorders, especially the influence of factors such as sex, lesion location, involvement of neuroendocrine factors and possible side effects related to DMTs. Finally, larger RCTs may shed light on the effectiveness of pharmacological therapy and psychotherapy in MS patients with neuropsychiatric symptoms.
